# Human Botfly: A Case Report and Overview of Differential Diagnosis

**DOI:** 10.1177/2324709618801692

**Published:** 2018-10-07

**Authors:** Mina Shenouda, Garrett Enten, Thanh Nguyen, Devanand Mangar, Enrico Camporesi

**Affiliations:** 1 TEAMHealth Research Institute, Tampa, FL, USA; 2 Memorial Hospital, Tampa, FL, USA

**Keywords:** *Dermatobia hominis*, human botfly, furuncular lesion

## Abstract

*Dermatobia hominis*, commonly known as the human botfly, is native to Tropical America. As such, cutaneous infestation by its developing larvae, or myiasis, is quite common in this region. The distinct dermatological presentation of *D hominis* myiasis allows for its early recognition and noninvasive treatment by locals. However, it can prove quite perplexing for those unfamiliar with the lesion’s unique appearance. Common erroneous diagnoses include the following: folliculitis, benign dermatocyst, and embedded foreign body with localized infection. We present a patient who acquired *D hominis* while she was in Belize. In this report, we discuss the presentation, differential diagnosis, diagnostic tests, and therapeutic approaches of human botfly lesion to raise the awareness about human botfly.

## Introduction

Skin disorders are among the most common medical consequences of short visits to developing countries.^1^ Myiasis or cutaneous infestation by developing larvae (Greek, myia = a fly) is the fourth most common travel-associated skin disease.^2^ *Dermatobia hominis*, commonly known as human botfly, is found in Central and South America, from Mexico to Northern Argentina, excluding Chile.^3^ We present the case of a patient who acquired *D hominis* while she was on a short vacation in Belize. In this report, we discuss the presentation, differential diagnosis, diagnostic tests, and therapeutic approaches of human botfly lesion to raise the awareness about human botfly.

## Presentation

**Figure 1.**
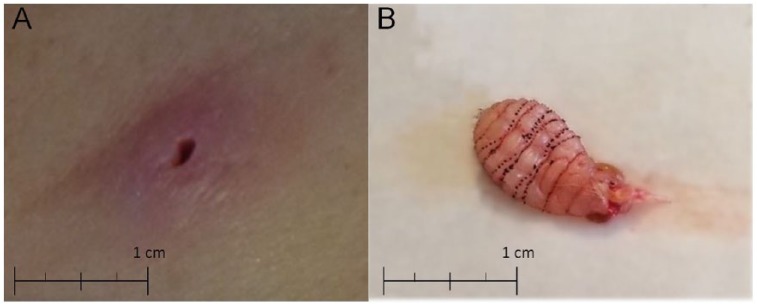
(A) Furuncular lesion of *Dermatobia hominis* contains a deeply embedded maggot: an indurated papule with a central opening, which allows the larvae to breathe. (B) *Dermatobia hominis* larva: note the tapered shape with rows of spines to prevent simple extrusion.

The patient is a 36-year-old female, presenting to the Department of Internal Medicine in Tampa General Hospital, reporting a lesion in her left inguinal area that she noticed 2 months prior after returning from her trip to Belize. She stated that she might have been bitten by an insect; however, she was unsure. While she was in Belize, she went horseback riding after which she found a tick on her back. She removed the tick immediately and states that it was present on her for no more than an hour. Once she returned to the United States, she went to her previous primary care physician who prescribed antibiotic, sulfamethoxazole. She completed the antibiotic course, but only saw a 50% improvement in the erythema surrounding the lesion without papule resolution. She requested her medical care to be transitioned to Tampa General Hospital where she reported the lesion (Figure 1A) initially had surrounding erythema, which has resolved. However, there was an indurated papule with a central opening that expressed opaque fluid on manipulation. The patient reported that it was previously pruritic. She denied previous similar lesion, contact to similar lesion, rashes, fevers, chills, bleeding, fatigue, or any other new-onset symptoms. Possible cyst, infectious, or foreign body/ingrown hair were considered as differential diagnoses. Previous attempt to remove suspected foreign body or hair has been unsuccessful. The internist referred the patient for a dermatologist for consultation and further evaluation.

This consultation was unsatisfactory as the patient independently sought a second opinion from our wound care facility at Memorial Hospital. Reexamination of the lesion by a wound care specialist yielded evidence of a small hard mass underlying the papule on palpation. With this new finding, the patient was referred to a general surgeon for mass extraction. Following local anesthesia, a 5 mm surgical incision across the lesion was made and the suspected foreign object was removed (Figure 1B). The foreign object was subsequently sent to pathology for identification and analysis. Pathology identified the object as a human botfly larva. With the larva removed, the lesion completely resolved by the patient’s follow-up visit at the wound care facility a week later.

## Discussion

Myiasis caused by *D hominis* is rarely seen in the United States. However, it is very common among residents and visitors of the tropical regions of the Americas.^4^ The female human botfly lays her eggs on the body of an intermediate host (eg, a mosquito or a fly), which acts as a vector onto the human skin when it feeds.^5^ The heat of the skin causes the eggs to hatch into larvae, which breathe through a central punctum. Unlike other family members of botflies, the larva of the human botfly does not migrate far into the skin from its point of entrance.^6,7^ The larval stage in the skin tissue can last between 27 and 128 days before the adult larva drops to the ground where it pupates for between 27 and 78 days before maturing into an adult botfly. The adult form of the human botfly is rarely seen and ranges between 1 and 3 cm long. The whole life cycle lasts between 3 and 4 months.^8^

Furuncular myiasis, caused by *D hominis* larvae, presents as a hard raised lesion in the skin with central necrosis—sometimes painful and pruritic. It mostly affects the limbs, though presentation on the genitals, scalp, breast, and eye has been reported.^9-12^ In some cases, the patients can feel the larvae moving when they shower or cover the wound.^13^ Because of the unspecific symptoms and low incidence of *D hominis* in the United States, misdiagnosis or delayed treatment of the lesion happens frequently as our patient experienced. High clinical suspicion is needed to diagnose these types of lesions.

Diagnosis is confirmed by identification of fly larvae or maggots. However, complete blood cell count may show elevated levels of leukocytes and eosinophils as the presence of the larva in the skin often triggers a local inflammatory response with the migration and proliferation of inflammatory cells including neutrophils, mast cells, eosinophils, fibroblasts, and endothelial cells.^14-16^ Imaging may be needed for patients with atypical presentations in unusual anatomical sites.^17^ Magnetic resonance imaging can be used in cases of cerebral, breast, facial, orbital, and furuncular myiasis. Computed tomography scan has also been suggested.^18^ Additionally, ultrasonography is very useful in establishing the diagnoses and in determining the size of the larvae. Quintanilla-Cedillo et al^19^ have described using Doppler ultrasonography utilizing a high-resolution (10-MHz) soft-tissue transducer. This allowed for early visualization of the larvae to confirm myiasis when lesions were small, had minimal secretions, and the punctum was absent, a point where the lesion can often be mistaken for a simple insect bite. In these cases, high-resolution Doppler ultrasonography was demonstrated to be both 100% sensitive and specific in diagnosis.

In a review of tropical myiases, McGarry^20^ stated that the slowly growing, often painful boil-like furuncular lesion of *D hominis*, which contains a deeply embedded maggot, requires surgical removal. However, most cases of *D hominis* do not require invasive surgery and can be treated by the patients themselves through noninvasive approaches. Local residents in Belize suffocate the larvae by applying occlusive substances,^21^ for example, placing petroleum jelly, bacon strips, nail polish, or plant extracts over the central punctum. Several hours after occlusion the larvae will emerge head-first seeking air, at which time, tweezers may be used to physically extract it or apply pressure around the cavity aiding in the larvae expulsion. Generally, larvae emerge 3 to 24 hours after application of the occluding agent.^22,23^ Despite the fact that these approaches affect the oxygen requirements of the larvae and encourage migration on their own, sometimes the larvae suffocate without migration from the skin and this might lead to incomplete extraction and secondary infection that precipitate surgical removal.

Surgical removal with local anesthesia is the treatment of choice for *D hominis* lesion. During surgical removal, a local anesthetic is applied and the lesion is excised; the wound is then debrided of remaining necrotic tissue and closed. This achieves complete removal of the larvae and prevents a secondary infection. Another method involves the injection of lidocaine into the base of the lesion. This creates a buildup of fluid pressure that forces the larvae out of the punctum.^24^ Alternatively, a 4- to 5-mm punch excision of the overlying punctum and surrounding skin may be performed. This grants greater visibility and better access to the larva, which can then be removed using toothed forceps.^25^ These methods are necessary as the larva cannot be removed through the central punctum; the larvae have evolved a tapered shape and rows of spines and hooks that prevent simple extraction. If attempted, simple extraction may result in retained portions of the larvae resulting in infection or an inflammatory reaction. Whichever technique is applied, it is recommended to thoroughly clean the resulting wound and apply an antiseptic dressing. Should the procedure result in a secondary infection, oral or topical ivermectin (1% solution) is indicated. Although it is quite rare to see cases of botfly lesions in the United States, their accurate diagnoses and early treatment is critical to avoid any possible complications or mistreatment.
